# Supplementary material for: A De Novo DNM1L Mutation in Twins with Variable Symptoms, Including Paraparesis and Optic Neuropathy
Source: Biomolecules. 2025 Aug 26;15(9):1230. doi: 10.3390/biom15091230 (PMC12467091; doi:10.3390/biom15091230)
Supplement: Supplementary file 1 [file biomolecules-15-01230-s001.zip › biomolecules-3778089-supplementary.pdf]

Table S1 TMPs for all individual *DNM1L* transcripts

Gene	Transcript ID	Ct1	Ct2	Ct3	Ct4	Ct5	Ct6	Ct7	Ct8	Ct9	Ct10	Ct11	Pt2	CCDS	AA	RefSeq	DALTON	%Pt2 vs Cts
DNM1L	**ENST00000553257.6**	0	0	0	0	0	0	0	0	0	0	0	0	CCDS61095	749	NM_001278464.2	82390	**0%**
DNM1L	**ENST00000381000.8**	0	7	0	5	0	0	6	3	1	0	2	0	CCDS61096	738		81180	**0%**
DNM1L	**ENST00000547312.5**	0	0	0	4	6	3	0	0	0	5	0	0	CCDS61098	725		79750	**0%**
DNM1L	**ENST00000358214.9**	0	0	0	0	0	4	0	0	0	0	0	0	CCDS81680	712		78320	**0%**
DNM1L	**ENST00000552743.1**	0	0	0	0	0	0	0	0	0	0	0	0		no protein			**0%**
DNM1L	**ENST00000550154.5**	0	0	0	0	0	0	0	0	0	0	0	0		168		18480	**0%**
DNM1L	**ENST00000547548.1**	0	0	0	0	1	0	0	0	0	0	0	0		no protein			**0%**
DNM1L	**ENST00000546649.5**	0	0	0	0	0	0	0	0	0	0	0	0					**0%**
DNM1L	**ENST00000413295.6**	0	0	0	0	0	0	0	0	0	0	0	0					**0%**
DNM1L	**ENST00000452533.6**	9	16	8	11	14	21	14	13	13	16	23	10	CCDS8730	710		78100	**73%**
DNM1L	**ENST00000553031.1**	0	3	0	0	1	1	2	1	1	0	1	1		no protein			**112%**
DNM1L	**ENST00000266481.10**	10	6	6	15	4	8	17	10	5	5	8	10	CCDS8728	699		76890	**117%**
DNM1L	**ENST00000549701.6**	9	0	5	3	9	4	0	6	4	0	0	7	CCDS8729	736	NM_012062.5	80960	**182%**
DNM1L	**ENST00000547640.1**	1	5	3	2	5	1	4	3	4	0	1	5		no protein			**183%**
DNM1L	**ENST00000548151.1**	1	1	1	1	1	0	4	1	0	0	0	3		no protein			**337%**
DNM1L	**ENST00000549157.1**	0	0	0	0	0	1	0	1	1	0	1	4		no protein			**985%**

Quantification counts of individual transcripts of all ENST related to the DNM1L gene from eleven controls (Ct 1-11) and patient 2 (Pt2).
The last column (%Pt2 vs Cts) indicates in percentage the abundance of ENST in patient 2 compared to the average of the controls.
CCDS consensus coding sequence; AA aminoacids; «no protein» transcript that does not produce protein.
