# Supplementary figures and images for: A De Novo DNM1L Mutation in Twins with Variable Symptoms, Including Paraparesis and Optic Neuropathy
Source: Biomolecules. 2025 Aug 26;15(9):1230. doi: 10.3390/biom15091230 (PMC12467091; doi:10.3390/biom15091230)

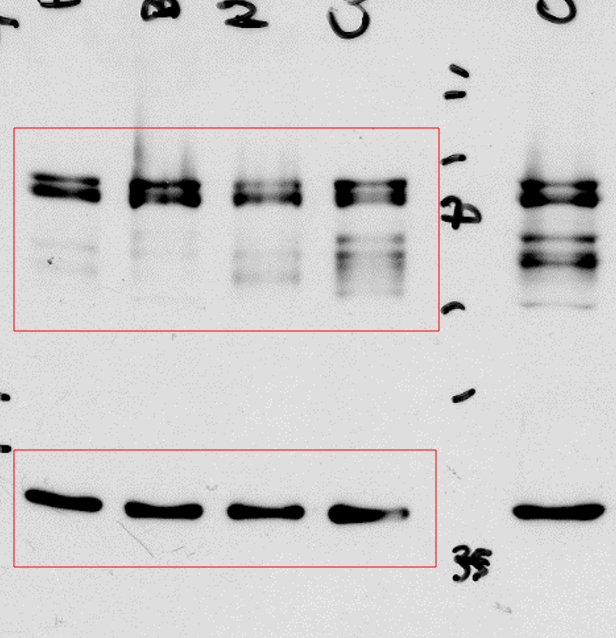

Supplement: Supplementary file 1 [file biomolecules-15-01230-s001.zip › biomolecules-3778089-WB/dnm1l_gapdh.tiff]

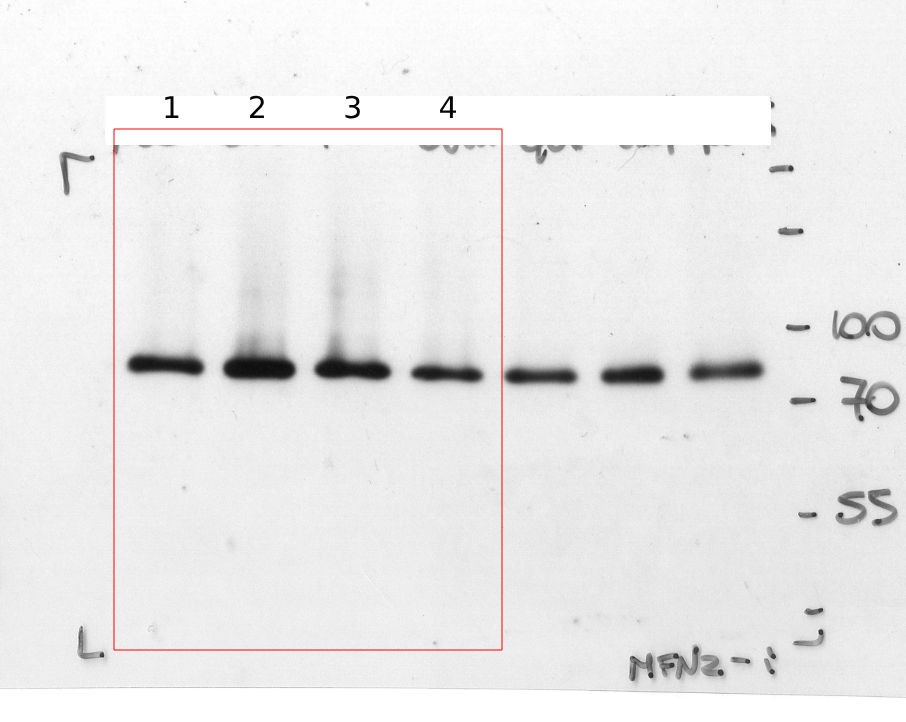

Supplement: Supplementary file 1 [file biomolecules-15-01230-s001.zip › biomolecules-3778089-WB/mfn2.tiff]

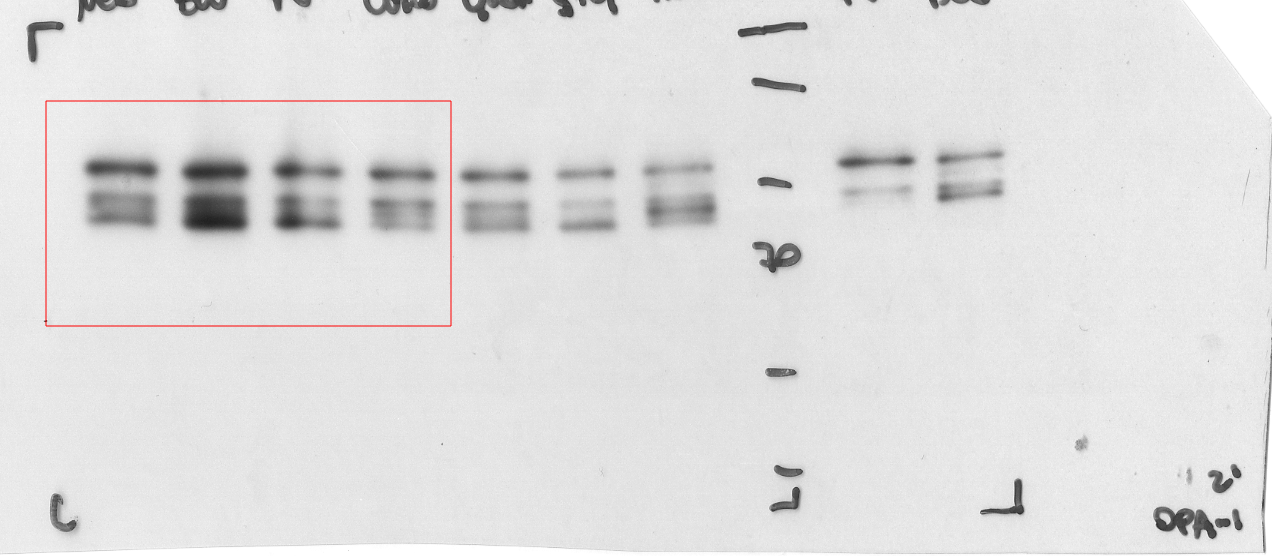

Supplement: Supplementary file 1 [file biomolecules-15-01230-s001.zip › biomolecules-3778089-WB/opa1.tiff]

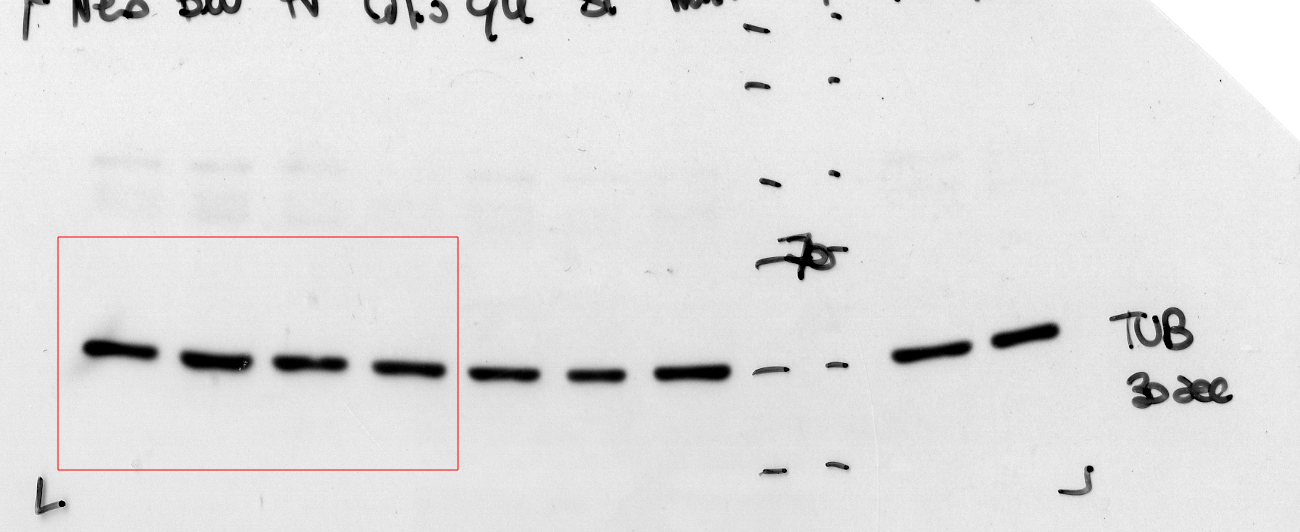

Supplement: Supplementary file 1 [file biomolecules-15-01230-s001.zip › biomolecules-3778089-WB/tubulin.tiff]
